# Image‐fusion‐guided focal irreversible electroporation for prostate cancer: Step‐by‐step technique

**DOI:** 10.1002/bco2.70266

**Published:** 2026-08-12

**Authors:** Masatomo Kaneko, Yuta Inoue, Federico Eskenazi, Andre Luis Abreu

**Affiliations:** ^1^ Center for Image‐Guided Surgery, Focal Therapy and Artificial Intelligence for Prostate Cancer USC Institute of Urology, University of Southern California Los Angeles California USA; ^2^ Department of Radiology, Keck School of Medicine University of Southern California Los Angeles California USA

**Keywords:** image fusion, irreversible electroporation, magnetic resonance imaging, partial gland ablation, prostate biopsy, prostate cancer

## 1 INTRODUCTION

Irreversible electroporation (IRE) is a non‐thermal ablation for prostate cancer (PCa) that uses high‐voltage electrical pulses to disrupt cell membranes and induce targeted cell death while potentially eliciting a PCa‐specific systemic immune response.^1^, ^2^ The procedure is typically performed under transrectal ultrasound (TRUS) guidance. Fusion of preoperative prostate magnetic resonance imaging (MRI) with real‐time TRUS may facilitate precise lesion targeting while preserving urogenital function.^3^

To date, no educational paper has described in detail image‐fusion‐guided focal IRE for PCa. Herein, we describe our step‐by‐step technique for MRI/TRUS image‐fusion‐guided focal IRE for PCa, along with the outcomes of a case series.

## 2 PATIENTS AND METHODS

### 2.1 Study population

Prospective patients undergoing multiparametric (mp) prostate MRI, followed by MRI/TRUS image‐fusion‐guided focal IRE (image‐fusion‐guided IRE) for primary localized PCa from November 2023 to May 2026, were evaluated (IRB # HS‐17‐00749). Exclusion criteria were (1) prior whole‐gland or systemic treatment for PCa, (2) no baseline mpMRI and (3) suspected metastatic disease.

At baseline, mpMRI was performed and interpreted according to the Prostate Imaging Reporting & Data System (PI‐RADS) version 2.1 by an experienced radiologist (≥500 prostate mpMRI interpretations per year).^4^ A baseline prostate three‐dimensional (3D) biopsy (PBx), consisting of 12‐core systematic sampling and ≥3 targeted cores from each PI‐RADS ≥3 lesion, was performed to histologically confirm the location of the index lesion (the highest Gleason Grade Group [GG] with the largest volume).^5^

Contraindications to IRE include epilepsy, cardiac arrhythmia and recent myocardial infarction.

Follow‐up evaluations included assessment of prostate‐specific antigen (PSA) levels and urogenital symptoms at 3‐month intervals during the first year and at 6‐month intervals thereafter. MRI is performed annually. Follow‐up prostate biopsies (FU‐PBx) were conducted at 1 year and subsequently as clinically indicated.^4^

### 2.2 Step‐by‐step technique

The procedure is performed under general anaesthesia with neuromuscular blockade to minimize muscle spasms induced by high‐voltage pulses (Video S1). The patient is placed in the lithotomy position, and a urethral catheter is inserted.

The IRE system (NanoKnife, AngioDynamics Inc., Queensbury, NY) is used with 19‐G electrodes. For treatment planning, an MRI/TRUS fusion device (Trinity, Koelis, Grenoble, France) with a side‐fire endocavity TRUS probe is used to overlay biopsy tracks and MRI tumour contours onto real‐time ultrasound.^6^ The probe is secured in a holder, and a grid template guides precise electrode placement.

The first step is 3D TRUS registration. Following intraoperative TRUS acquisition, baseline biopsy cores and lesion coordinates are displayed on the fusion console. The urethra and the prostate surface are segmented, after which the biopsy cores and lesion are elastically fused to the TRUS images to visualize the 3D distribution of PCa.

**FIGURE 1:**
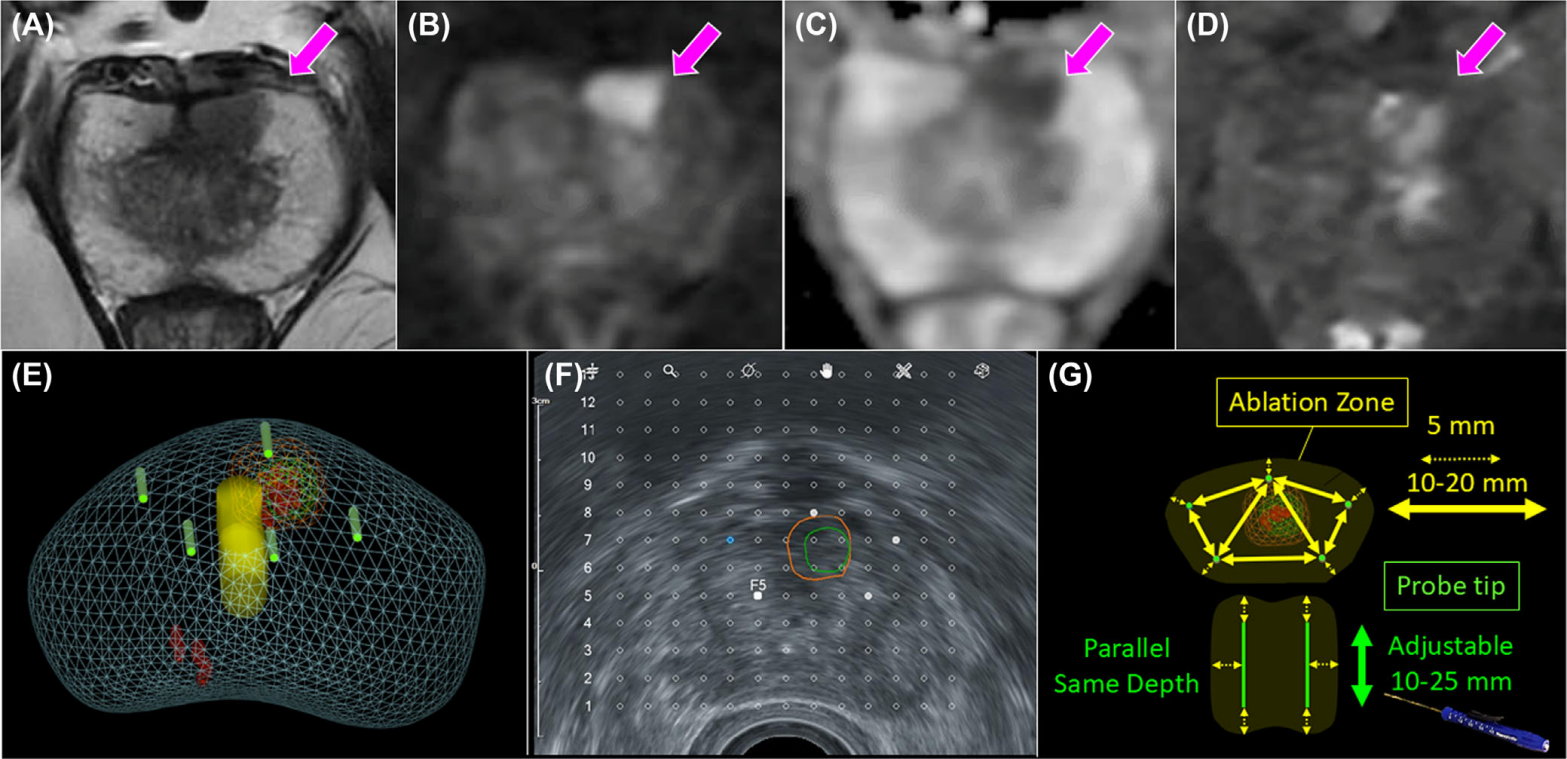
Representative case of image‐fusion‐guided irreversible electroporation. At baseline, the prostate multiparametric MRI ([A] T2‐weighted imaging, [B] diffusion‐weighted imaging, [C] apparent diffusion coefficient map and [D] dynamic contrast‐enhanced imaging) demonstrated a 43 cc gland with a PSA density of 0.1 ng/mL^2^ and a 13 mm PI‐RADS 4 lesion (pink arrow) in the left mid‐anterior prostate. (E) MRI/TRUS fusion‐guided transperineal prostate biopsy revealed GG 2 clinically significant prostate cancer localized to the left mid‐anterior lesion (red cores in the left lobe). A right‐sided systematic core demonstrated a small‐volume GG 1 insignificant cancer (red cores in the right lobe). (F) The IRE electrode placement through a grid template. The orange sphere indicates the MRI‐defined target boundary, whereas the green sphere represents the TRUS‐defined boundary. (G) Key considerations regarding treatment extent and spatial relationships during IRE electrode placement. GG, Gleason grade group; IRE, irreversible electroporation; MRI, magnetic resonance imaging; PI‐RADS, Prostate Imaging Reporting and Data System; PSA, prostate‐specific antigen; TRUS, transrectal ultrasound.

Before electrode insertion, the electrode distribution is virtually planned on the image‐fusion console (Figure 1). In IRE, electric pulses are delivered between electrodes to ablate tissue. The ablation zone extends approximately 5 mm beyond the electrode and is concentrated along the exposed tip, which is adjustable from 10 to 25 mm. Interelectrode spacing should be 10–20 mm. The electrodes should be placed in parallel and aligned at the same depth.

By matching anatomic landmarks between the virtual scan and the real‐time TRUS, electrodes are transperineally placed at the target location. The exposed tip must remain entirely within the prostate. To preserve urinary function, the urinary sphincter should be avoided.

After placement, all electrodes are connected to the IRE device. Typical settings include using two to six electrodes, depending on target size. Voltage ranges 500–3000 V, with 1.0–2.0 cm interelectrode spacing and a voltage‐to‐distance ratio of 1500 V/cm. Each electrode pair receives 90 pulses (range, 70–90) at 90 μs (range, 70–90 μs) and 90 pulses/min.

Of the 90 pulses, the first 10 are delivered as a conductivity test. Although the therapeutic range is 20–50 A, a target range of 20–35 A is typically selected because current increases during treatment. The active electrode pair and corresponding currents are displayed on the monitor. Small electrode movements may occur because of pulse‐induced muscle contractions. If the current deviates from the desired range, the voltage is adjusted accordingly.

After voltage adjustment, the remaining 80 pulses are delivered. Current may increase because of tissue oedema, hydrolysis, Joule heating and electroporation. Currents >50 A increase the risk of thermal injury, whereas currents <20 A may result in under‐treatment; therefore, careful monitoring is required. Concurrently, ultrasound is used to confirm the development of a hyperechoic ablation zone, indicating effective treatment.

The procedure typically lasts 1.5 h with estimated blood loss <5 mL. A Foley catheter is maintained for 1 week to prevent acute urinary retention.

## 3 RESULTS

A total of 12 patients who underwent IRE were screened, and one patient was excluded due to the unavailability of mpMRI based on PI‐RADS. The median (interquartile range [IQR]) age, PSA, PSA density and follow‐up duration were 74 years, 6.86 ng/mL, 0.14 ng/mL^2^ and 11 months, respectively. The baseline GG distribution was 9.1% GG1 and 91% GG2, corresponding to 9.1% low‐risk and 91% intermediate‐risk disease. PI‐RADS scores were 3 in 36% and 4 in 64% of the patients.

After IRE, median PSA decreased by 70% to a nadir of 2.0 ng/mL among patients with available follow‐up PSA (8/11). During follow‐up, treatment failure (GG ≥ 2 on FU‐PBx, salvage whole‐gland/systemic treatment, metastasis or PCa‐specific mortality)^4^ was observed in 18% (2/11); both cases were GG ≥ 2 on FU‐PBx and were managed with active surveillance. The FU‐PBx rate was 45% (5/11). No biochemical failure (PSA nadir + 2 ng/mL) (0/8), radical treatment (0/11), metastasis (0/11) or PCa‐specific mortality (0/11) was observed.

Regarding functional outcomes, the median change in International Prostate Symptom Score (IPSS) and the International Index of Erectile Function‐5 (IIEF‐5) scores from pre‐ to post‐IRE were 1 and 3, respectively.^6^ Continence (zero‐pad) was maintained in 100%. Ninety‐day postoperative complications occurred in 27% (3/11), including Clavien grade 1 urinary tract infection and grade 1 and 2 urinary tract retentions.^6^

## 4 DISCUSSION

This step‐by‐step video provides a practical overview of image‐fusion‐guided IRE and serves as an educational resource for clinicians adopting this technique. In conjunction with our case series, the findings support the technical feasibility of image‐fusion‐guided IRE for predominantly MRI‐visible intermediate‐risk PCa, the typical target population of focal therapy,^7^ with favourable short‐term oncologic and functional outcomes.

Intraoperative TRUS guidance alone provides limited visualization of PCa, particularly for lesions anterior to the urethral catheter or obscured by calcifications, and remains highly operator dependent. MRI/TRUS image fusion enables visualization of biopsy‐proven cancer cores and MRI‐defined lesions, facilitating accurate and efficient probe placement while maintaining safe distances from critical structures.

The observed rate of GG ≥2 on follow‐up biopsy (18%) compares favourably with outcomes reported in prospective focal therapy studies, including contemporary IRE series.^1^, ^8^ Similarly, the overall complication rate (27%), grade ≥2 complication rate (9.1%) and absence of severe complications suggest a favourable safety profile.^1^, ^8^

Limitations of the study include a small sample size, short follow‐up associated with this new technique resulting in incomplete FU‐PBx in some patients and a cohort predominantly comprising MRI‐visible intermediate‐risk disease, which requires caution when extrapolating these findings to low‐risk or MRI‐invisible disease. Larger cohorts with longer follow‐up are warranted to validate these findings. Nevertheless, this detailed step‐by‐step video may help disseminate the technique and facilitate adoption of image‐fusion‐guided IRE.

## 5 CONCLUSION

Image‐fusion‐guided IRE appears feasible and demonstrates encouraging short‐term oncologic and functional results.

## AUTHOR CONTRIBUTIONS

**Masatomo Kaneko:** Conceptualization; methodology; data curation; investigation; formal analysis; visualization; writing—original draft; writing—review and editing. **Yuta Inoue:** Investigation; visualization; writing—review and editing. **Federico Eskenazi:** Investigation; visualization; writing—review and editing. **Andre Luis Abreu:** Conceptualization; methodology; resources; writing—review and editing; supervision; project administration.

## CONFLICT OF INTEREST STATEMENT

Andre Abreu is a proctor and speaker for Sonablate and EDAP and proctor and has a research grant with Koelis; has clinical trial with Francis; and is an unpaid consultant for Procept BioRobotics.

## ETHICS STATEMENT

All procedures in the study were conducted according to the ethical standards of the institutional (IRB number HS‐17‐00749) and/or national research committees, as well as the 1964 Declaration of Helsinki and its subsequent amendments or equivalent ethical guidelines. The data used in the study were retrieved from the Health Insurance Portability and Accountability Act (HIPAA) compliant prostate biopsy database.

## DISCLOSURE

During the preparation of this work, the authors used ChatGPT 5.2 Edu to correct English grammar. After using this tool, the authors reviewed and edited the content and take full responsibility for the content of the publication.

## Supporting information


**Video S1:** Step‐by‐step video of image‐fusion‐guided irreversible electroporation.

**Data S1.** Supporting Information.

## Data Availability

The data analysed during the current study are available from the corresponding author on reasonable request.
