# Supplementary material for: Image‐fusion‐guided focal irreversible electroporation for prostate cancer: Step‐by‐step technique
Source: BJUI Compass. 2026 Aug 12;7(8):e70266. doi: 10.1002/bco2.70266 (PMC13467490; doi:10.1002/bco2.70266)
Supplement: Supplementary file 1 [file BCO2-7-e70266-s001.docx]

**Transcript**

Image-Fusion–Guided Focal Irreversible Electroporation for Prostate Cancer: Step-by-Step Technique

The following is a 74-year-old male with an elevated PSA of 4.3.

The prostate multiparametric MRI demonstrated a 43cc gland with a PSA density of 0.1, revealing a 13mm PIRADS 4 lesion in the anterior horn of the left mid prostate. This raised concern for possible clinically significant prostate cancer.

An MRI, Transrectal Ultrasound fusion, Transperineal prostate biopsy was performed, revealing grade group 2 clinically significant prostate cancer localized to the left mid-anterior lesion. The right systematic core identified small grade group 1 insignificant cancer.

MRI‐visible, anteriorly located intermediate-risk prostate cancer is a good indication for image-fusion-guided irreversible electroporation (IRE). After careful discussion about the current standard of care, the patient underwent focal IRE.

IRE is an ablation technique that uses short, intense electrical pulses to disrupt cell membranes and induce cell death. Because it is a non-thermal modality, IRE is considered safe for preserving surrounding vital structures.

The procedure is performed under general anesthesia with deep neuromuscular blockade to minimize high-voltage–induced muscle spasms. The patient is placed in lithotomy position, and the perineum is prepped and draped in the standard sterile fashion. A urethral catheter is inserted.

The NanoKnife IRE system from AngioDynamics is used with 19-gauge electrode probes. In addition, a 3D ultrasound with a side-fire endocavity TRUS probe can be used to overlay biopsy tracks and MRI tumor contours onto real-time ultrasound, supporting precise treatment planning. The TRUS probe is secured with a probe holder, and the probe-mounted grid template guides the precise placement of the electrodes.

First, the intraoperative transrectal ultrasound is performed using a probe holder. The baseline biopsy cores and corresponding lesion coordinates are displayed on the transrectal ultrasound console, with cancer-positive cores highlighted in red and benign cores depicted in green. Next, the urethra is delineated on the intraoperative 3D TRUS images. The prostate surface is then segmented, and finally, the baseline TRUS is elastically fused with the intraoperative TRUS to visualize the three-dimensional distribution of the prostate cancer.

The orange sphere indicates the target lesion boundary determined by MRI, while the green sphere represents the boundary defined by ultrasound. Before actual needle insertion, the distribution of the IRE probes is virtually planned on the ultrasound fusion console. To ensure complete coverage of the target lesion, probe locations are precisely determined using a brachytherapy grid template.

In IRE, electric pulses are delivered between the probes to ablate prostate tissue. The ablation effect extends approximately 5 millimeters beyond the probe surface and is concentrated along the exposed tip, which is adjustable between 10 and 25 millimeters. The distance between the probes must be set between 10 and 20 millimeters. The probes should be placed in parallel and aligned at the same depth. Contralateral, small, clinically insignificant prostate cancers may be left untreated.

By comparing anatomic structures between the virtual scan and the real-time TRUS image, and using the grid guide as reference, IRE probes are transperineally placed at the targeted location. It is then confirmed that the exposed probe tip are fully contained within the prostate.

To ensure urinary function and decrease the risk of incontinency, the urinary sphincter should not be ablated. The remaining IRE probes are placed with reference to the virtual probe positions.

After placement, all IRE probes are connected to the IRE device.

Typical ablation settings are shown. The number of electrodes depends on the size of the target, with up to six electrodes used. The initial voltage is determined by dividing the voltage between electrodes by the distance separating them, referred to as the voltage-to-distance ratio, which is typically set at about 1500 volts per centimeter.

Ten of the ninety pulses are first delivered as a conductivity test. While the therapeutic range is twenty to fifty amps, a lower target of twenty to thirty-five amps is used since current rises during treatment. The active pair flashes on the schematic, with actual currents shown in the table and waveforms. During IRE delivery, small movements of the electrodes are seen, caused by muscle contractions from the electrical pulses. Here, the test revealed higher than predicted current, indicating the need for voltage adjustment.

After voltage adjustment, the remaining eighty pulses are delivered. During this phase, amperage may rise because of tissue edema and inflammation, aeration from hydrolysis, temperature increase from joule heating, and the electroporative effect. If the current exceeds fifty amps, the risk of thermal damage to adjacent structures increases, so careful monitoring is required. At the same time, intraoperative ultrasound is used to confirm that the ablation zone appears as a hyperechoic area, indicating effective treatment.

Electric pulses are successfully delivered as planned.

The procedure was completed without intraoperative complications. The estimated blood loss was less than 5 mL. Operative time was only 68 minutes, and a Foley catheter was left for 1 week.

At the 3-month follow-up, a 72% reduction in PSA was achieved, indicating the treatment success. The patient is asymptomatic and continent, with two point improvement in QOL.

Image-fusion-guided focal irreversible electroporation is precise, safe, time-efficient, and has the potential to achieve adequate prostate cancer control.

Thank you.
